# Incorporating community perspective in research funding: creation of the National Multiple Sclerosis Society Community Review of MS Research Committee

**DOI:** 10.1186/s40900-025-00686-3

**Published:** 2025-02-24

**Authors:** Anna T. Lampe, Kathleen M. Zackowski

**Affiliations:** National Multiple Sclerosis Society, 733 3rd Avenue, 3rd Floor, New York, NY 10017 USA

## Abstract

Integrating community voices in scientific research is important to ensure the research outcomes reflect what is meaningful to that community. However, the process by which individuals from the community are included in scientific grant funding decisions is variable and often comprises only a patient representative on a review panel or advisory board that is largely composed of scientific experts in the field. Finding the right balance for incorporating and honoring the scientific community’s technical expertise and the patient community’s lived expertise has been a growing discussion. The work of the National Multiple Sclerosis (MS) Society is a movement by and for all people affected by MS, meaning that the perspectives of people living with MS (MS community) are essential in all work conducted by the Society. In alignment with this, the National MS Society formed the Community Review of MS Research Committee (Committee) in 2021 to gather feedback from MS community members on research grant applications. The Committee’s feedback focuses on patient-centered concerns and relevance to the MS community. The Committee is composed of a diverse group of non-scientist individuals affected by MS that provide the Society with unique MS community-specific feedback, separate from the established scientific review committee. Here the process of identifying the goals of this Committee and planning of the processes used by the committee, as well as committee implementation, execution, and refinement over three years is described. Through feedback from this Committee, it was demonstrated that people affected by MS have important perspectives and insights into research projects focused on MS and can offer unique guidance to scientist applicants. This Committee has provided benefits to the Society, the MS community, and the Committee members themselves. In addition, the formation of this Committee has allowed the Society to be better informed about topics that are relevant to the MS community and to comprehensively and systematically incorporate the MS community’s perspectives in research grant funding decisions. This publication is intended to share learnings from the development of this Committee to be useful for other disease communities with the goal of incorporating the community voice into scientific research.

## Abstract (plain language summary)

Including the voices of people from the community in scientific research is important to ensure that the outcomes of the research are meaningful to people in that community. Finding the right balance of the science and community voice in research grant application review has been a growing discussion. The National Multiple Sclerosis (MS) Society is a movement by and for all people affected by MS. This means that the perspectives of people living with MS (MS community) are essential to the work the Society conducts. To meet this goal, the National MS Society formed the Community Review of MS Research Committee (Committee) to gather feedback from MS community members on research grant applications. The Committee is a diverse group of non-scientists who are affected by MS and provide feedback focused on patient concerns and importance to the MS community, separate from the scientific review process. Here we describe the process of identifying goals and planning processes used by the committee. Through feedback from this Committee, we show that people affected by MS have important perspectives and insights into research projects focused on MS and can offer unique guidance to scientists, the Society, and Committee members. The formation of this Committee has allowed the Society to be better informed about topics that are important to the MS community and to include the MS community’s perspectives in research grant funding decisions. This information is intended to inform other communities who have a similar goal of incorporating the community voice into scientific research.

## Introduction

When studying a condition that affects people’s lives, such as multiple sclerosis (MS), it is critical to incorporate the community that is affected by the condition early in the research pipeline to ensure the research outcomes reflect what is meaningful to that community and ultimately improve clinical management [1]. In alignment with this, the work of the National Multiple Sclerosis (MS) Society (the Society) is a movement by and for all people affected by MS, meaning that the perspective of people living with MS is an essential voice in all work conducted by the Society. A key aspect of the Society’s work to cure MS while empowering people affected by MS to live their best lives is to award grant funding to studies in the field of MS. Therefore, it is at the center of the Society’s mission to incorporate the perspectives of people living with MS into the research grant decision-making process in a meaningful way.

The Society solicits applications for grant funding from researchers in the field of MS. Grant applications submitted to the National MS Society undergo multiple rounds of review before funding decisions are made. Historically this involved review by Society staff and outside expert peer reviewers in the field. The peer review process provides a review of the scientific aspects of the proposed research project. However, this previous system did not formally include a review of the proposed projects by a committee of people affected by MS. The Society set a goal to more meaningfully and systematically incorporate the perspectives of people affected by MS through a review committee composed entirely of those affected by MS, representing the MS community, which would parallel the scientific review process.

Upon review of processes used by other granting agencies, it was found that many other granting agencies did incorporate the perspective of patient stakeholders in their grant application review processes. However, this was often in the form of a patient representative participating on a review panel or advisory board that was largely composed of scientific experts in the field. Following a review of the advantages and disadvantages of this approach and other ways of incorporating lived experience, the Society set out to create a review committee composed entirely of those affected by MS that was additive to the scientific review committee process while being integrated into the Society’s wider review process. This builds an environment for the community reviewers where they are the experts on the lived experience and there are no scientific experts in the room to which community members might feel an obligation to defer. The Community Review of MS Research Committee (Committee) provides feedback on applications that have been determined by the scientific review committee to be scientifically sound and meritorious of support, focusing on patient-centered concerns and relevance to the MS community.

**Fig. 1:**
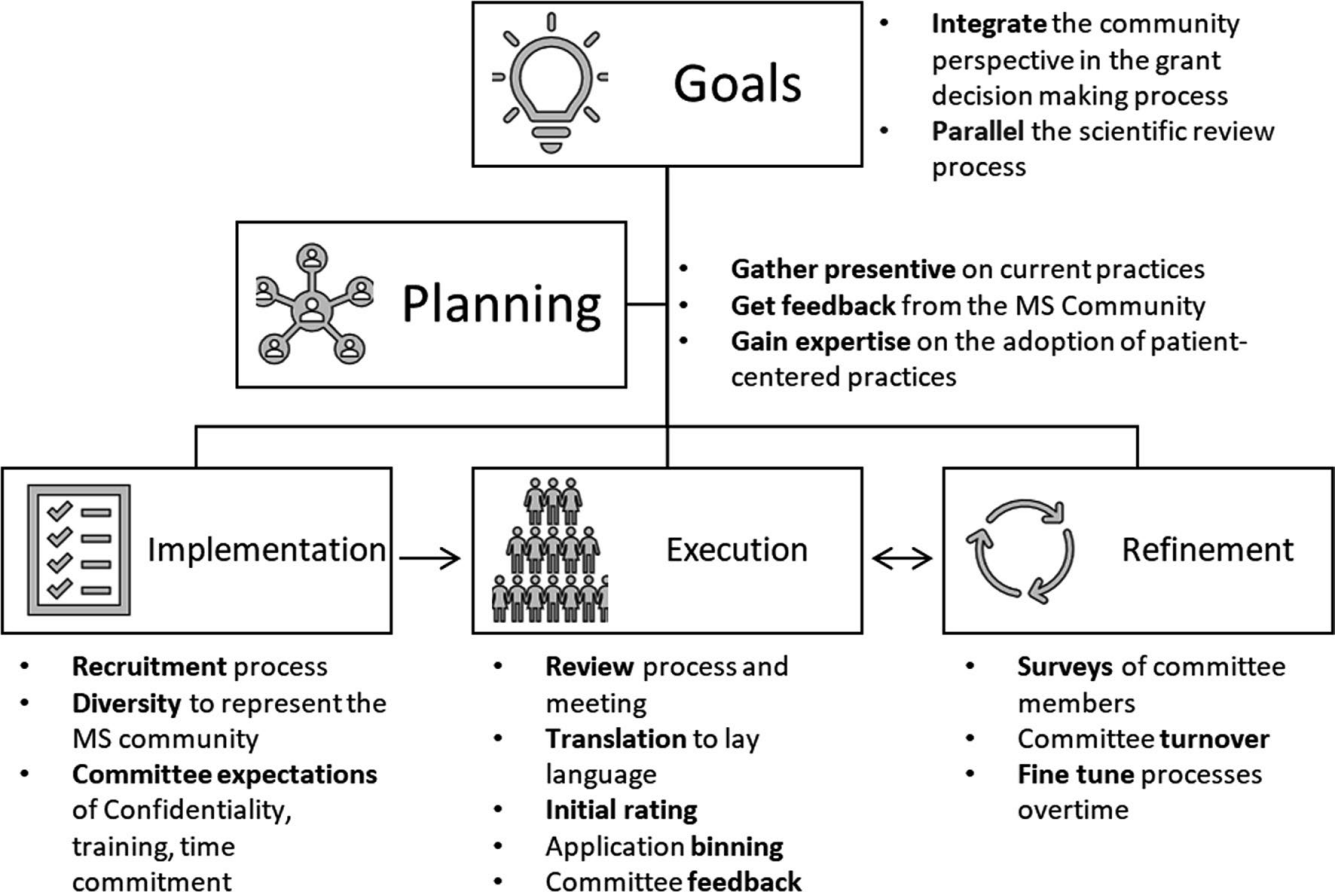
Overview of the planning and implementation of the Community Review of MS Research Committee

Here the process of identifying the goals of this committee and planning of the processes used by the committee, as well as committee implementation, execution, and refinement over time is described (Fig. 1). The goal of this publication is to disseminate the learnings from the creation of the Community Review of MS Research Committee that provides the perspective of people affected by MS for grant funding decision-making, and to inspire other organizations to incorporate the perspective of the affected community into research decision-making processes.

## Planning

As a first step, the Society evaluated existing literature and guidance on patient involvement in research. Society staff consulted with staff at other MS Societies globally, government funders and foundations inside and outside of the MS field. Society staff inquired about how other organizations incorporate the community into their research grant decision-making process and any learnings they could relay from their experience. This information provided an understanding of how other funders approach this issue of patient involvement in their review processes.

The Society also convened a Community Engagement in Research Task Force, a group inclusive of Society staff, scientific researchers (representing both clinical and basic research), MS clinicians, and people living with MS. The task force identified four goals for their work:

1. Identify the benefits of improving MS community engagement in the research grant decision-making process
2. Develop principles for how to incorporate MS community reviewers in the grant review process,
3. Develop strategies to incorporate MS community reviewers into the review meeting
4. Develop strategies for the sustainability of MS community engagement in the review process.

To meet these goals, the task force listened to and learned from each member's perspective on their experiences with the research review process. As a group, they synthesized information into critical considerations necessary for a revised research decision-making process. Key elements included establishing the new Community Review of MS Research Committee that will meet outside of the scientific review Committee and will be comprised of people affected by all types of MS. The committee was designed to be separate from the scientific committee to create a space where the discussion can be focused on the lived experience instead of including community members in a scientific discussion which has the potential to draw the focus away from the lived experience. It was deemed that this new Committee would use a parallel process to the scientific review committee. The Committee will have access to the entire application but will focus on the Plain Language Description provided by applicants. Committee members will score the application based on patient-centered concerns and relevance to the MS Community. The process was designed to be parallel and supplementary to the scientific review process to allow for the two committees to provide different types of feedback, which do not conflict or compete with feedback from the other committee. The scientific committee provides feedback related to the scientific merit of the proposed projects while the Committee gives feedback related to patient-centered concerns with study conduct and protocols and their perspective of the relevance of the work to the MS community. Neither committee provides funding recommendations to the Society, instead each committee provides separate types of feedback that are considered together by Society staff along with Society priorities and funding availability to make final funding recommendations to the CEO, who makes final funding decisions.

To prepare to implement the new goal of more comprehensively integrating the MS community into the Society’s research decision-making process, outside consultants who specialize in patient-centered practices were brought in. The Society initiated a formal relationship with the Kith Collective, a consulting practice specializing in helping organizations design and adopt patient-centered practices (https://www.kithcollective.com/). The Kith Collective helped integrate the planning information that was gathered by the task force and worked with Society Staff to create selection criteria and training modules for the new Committee with special attention focused on consensus- and team- building. This was important because this new Committee would have to work together to reflect MS community perspectives as broadly as possible.

As with any new process it took time to identify the goals of this new Committee, gather information on current practices, and gather feedback from the scientific and non-scientific MS community. From idea generation to identification of goals, these steps unfolded over a period of 18 months, but this timeline could be modified based on the needs of the community and available resources. From this initial process, as well as disruptions to normal operation due to the COVID-19 pandemic, it became clearer that implementation of the goals would require even more time. New timelines were set with the plan to implement the new Committee within the following 12 months**.**

## Implementation

### Committee member recruitment

The first step to implementing the Committee was to recruit those affected by MS to serve on the Committee. A recruitment process was created, building on the longstanding process that the Society had implemented with the scientific review committees and existing standing operating procedures for recruiting members to other Society volunteer advisory committees. Since the Committee would be expected to read and maintain confidential information from scientist applicants and be responsible for attending and participating in meetings, it was important that members have a history of volunteering with the Society to be nominated to serve on the Committee. This was one among a list of competencies that were identified as important for Committee members to possess (Table 1). Nominations were solicited through a presentation to the Society’s Senior Leadership team, asking for nominations of external individuals with previous and reliable Society volunteer experience, and who might have an interest in reading and providing input on research applications. A pre-screening process was created that included a brief conversation between Society staff and the nominator and if deemed appropriate, the next step was the completion of a written nomination form by the nominator and nominee. Once the nomination form was submitted, the Society staff leading the Committee scheduled a virtual meeting with the nominee. Using a prepared set of questions, Society staff conducted an interview-style discussion with nominees and answered any questions the nominee had about the Society, the Committee, and its work (Table 2). After all interviews were completed, Society staff selected Committee members based on the goal of achieving a diverse group of individuals, across multiple areas of diversity.

**Table 1:** Competencies desired for members of the Community Review of MS Research Committee

Key competencies
Living with MS or experience caring for a person with MS
Strong communication skills (verbal and written)
Comfortable representing the broader MS experience – your personal experience as well as that of others living with MS
Confident and respectful contributor in group discussions
Comfortable asking questions when information is unclear or complex
Comfortable with computers and associated technology for virtual meetings and electronic application review
Demonstrated interest and basic understanding of the MS research field (not a trained scientist)
Demonstrated commitment to Society’s research priorities
Willingness and ability to complete work on the projected schedule
Willingness and ability to travel up to once annually

**Table 2:** Questions used during recruitment conversation with Nominees

Discussion guiding questions
Please share with us a high-level overview of your experience with MS, starting with how you (or your loved one) came to be diagnosed and the most prominent effects of MS on your life
Your nomination by a member of the Society’s senior leadership speaks highly of the contributions you have made to the Society’s mission. What have you enjoyed most about other volunteer leadership opportunities with the Society? Is there anything you wish would have been different about those experiences?
Committee members will participate in a multi-part orientation to provide information about the Society’s research program and how the Committee will work independently and collectively to inform the decision-making process about specific grant applications. It would be helpful to hear what interests you most about the Society’s research program?
Participation on this Committee will be somewhat unique in that it will require a rather intense commitment for short periods of time and will be relatively inactive through other periods of the year. The expected time commitment each year is: Initial 10-h training and orientation for onboarding Approximately 20 h during each review cycle for independent review and scoring of applications (to take place once or twice a year). This will need to take place over a 2–3-week time period as it is just one part of a multi-step review cycle One committee meeting each review cycle that will be 6–8 h in length (or possibly broken into parts over two days). For now, those meetings will be held virtually, but we do anticipate that after COVID-related restrictions are lifted, the Committee will meet in person requiring travel and possibly an overnight stay Check-in teleconference meetings in between review cycles to share final funding decisions and updates for the next cycle What questions or concerns does this set of expectations raise for you?
Members of the Society’s Research Program staff will be partners to the Committee to address scientific and technical questions. We don’t expect members of this Committee to be experts on the scientific aspects, but we will depend on members to ask questions when information is unclear or too complex. With this being the first time, we’ve tapped community experience in this way, we will also be relying on Committee members to advise us on ways we can improve the process, including the training and the way we conduct the meeting to review applications. Please share an experience from your personal, professional, or volunteer activities where you have contributed to a similar process
What other questions do you have for me about the Society, our research program, this Committee, or the expectations? Is there anything more you’d like me to know about your interest in serving?

The Society took a thoughtful approach to assure diversity within the Committee. The overall goal was to represent as much of the MS community as possible with a small committee. Factors considered for this Committee included racial, ethnic, and gender diversity, type of MS, level of physical ability and factors such as geography, past volunteer experience with the MS community and the Society, and the individual MS journey. The distribution of these factors is reviewed annually when there is Committee member turnover. Additionally, Committee members are asked to reflect on what perspectives from the MS community may still be missing from the Committee during individual annual engagement conversationss. The Society is intentional about including important areas of diversity that may be identified from the Committee and the Society’s perspectives. During annual recruitment periods, Society leadership, committee staff support, and committee members consider any perspectives that may be missing from the committee and seek out those perspectives in recruiting new members.

### Committee member selection and maintenance

Seventeen nominations were collected in year one, and 13 Committee members were selected from that pool of nominees. Four nominees were not selected, in an effort to keep the size of the committee manageable. Fourteen committee members match well with the average size of the scientific review committees at the Society, small enough to manage logistics (i.e., the number of applications and scheduling) and large enough to be inclusive of a variety of viewpoints in the community. The four nominees not chosen to move on in the process represented overlaps or similarities among the nominees (i.e., from the same state with similar characteristics, similar age, etc.). Committee members were selected to join the Committee for one year, with the understanding that after each year, an engagement conversation would allow both the Society and the Committee members to decide if an additional one year of commitment was desired. After the inaugural Committee had completed about a year and a half of service, the first round of Committee member turnover was initiated. For the first round of Committee member turnover, three members rolled off and additional nominations were solicited from Society staff. Seven new nominations were received, and from those nominees, four new Committee members were selected to join the Committee, resulting in a total of 14 Committee members.

This larger Committee allowed for flexibility in the volume of applications to be reviewed in each cycle. For large review cycles requiring the review of 15 or more applications, the burden on the Committee was very high. To resolve this issue of high Committee burden, the Committee was split into two smaller committees, so each committee reviewed half of the applications. Staff divides the committee considering representativeness relative to the MS population so that each application receives comments from diverse MS perspectives. In smaller cycles, the group functioned as one large committee. In this way, each Committee would always have at least seven people with the MS lived experience reviewing applications for each cycle. A similar process is used with the Scientific review committees which use four smaller committees to review larger grant cycles.

### Committee training

Guiding the expectations of the Committee members was an important step in the implementation of this Committee. With the direction of the Kith Collective, basic training materials for the Committee members were created that included a document outlining the basic principles of the Committee (Table 3). Rules about confidentiality were explained and discussed with the Committee members, emphasizing the nature of the work they would be reviewing and the potential impacts of sharing this private scientific information outside of the Committee discussions. To formalize this process, Committee members review and sign a conflict-of-interest form annually, similar to what is done with the Scientific Committees. Prior to the three one-hour virtual onboarding training sessions, a set of newly created advanced training materials was distributed to the Committee. The advanced materials included information on the history of the Society, a review of the Society’s research priorities, the Committee's purpose and principles, an introduction to the training resources (including implicit bias training resources), a Zoom participant guide, and a review of the training session schedule. Training materials were created focusing on the use of plain language, assuming the reader has no scientific training. Materials were available with a variety of media and interactive training allowing for a question/answer approach. The goal was to give committee members the flexibility to pick the way they learn best. All materials are available electronically to allow more individual adaptability (i.e., they can increase font size, print them, etc.).

**Table 3:** Principles of the Committee

Principle	Description
Collectively, you are the voice of the MS community	You were selected to participate on the basis of your individual capabilities and for the way that together you form a representative composite of the broad and diverse MS community
You are an expert	Your lived experience as a person directly affected by MS provides you with a valuable perspective that is distinct from the expertise that a physician or scientist may have. You need not attempt to replicate their expertise. The Society has tailored the Community Review process to leverage your ability to make a unique contribution to the information that will be used to determine which research applications are funded by the Society
Strive to be impartial and open-minded	Remain alert to preconceptions you may hold about a researcher, institution, or research topic. Recuse yourself from a particular review if you may have a bias, whether it is positive or negative
Identify barriers to participating in or sticking with a study	You are asked to assess the feasibility and acceptability of study measures from the participant point of view. We hope this process will help to avoid participation challenges that can slow or stop a study and limit its value to understanding MS
Consensus is our goal	Your individual scores will determine where we focus time when the Committee meets together with the objective of reaching consensus about which grants are of greatest interest to the Community. Everyone’s perspective is valued and honored even if we don’t reach unanimous agreement
The Community Review of MS Research Committee is an advisory body	The guidance you provide at the conclusion of the Committee meeting will be fully considered and weighed in the final determination of which applications to approve for funding. Those decisions rest solely with the Society’s CEO. Your advice will also be sought for ways to improve the Community Review process, including recruitment, training and advance preparation, conduct of the review cycle, and communication at every step of the process
Maintain confidentiality	All reviewers involved in the Society’s research program are expected to hold the details of the research applications and the review discussions in strict confidence. This policy extends to the number of applications reviewed and the reasons why some are recommended for funding and others are not. There may be opportunities for you to share with the community about your general experience serving in this role in ways that don’t compromise details of the applications or deliberations

The first training session focused on basic introductions, sharing the Committee history and charge, reviewing a high-level view of the Society’s research review process, and establishing Committee meeting logistics (i.e., dates, times, etc.). The second training session focused on introducing the Society’s science and administrative teams, time for Committee members to share their individual MS journey experiences, consensus-building exercises, a review of rules of confidentiality and conflicts of interest, and instructions for how to access and score the grant applications on the Society online portal. Both the first and second training sessions ended with time for questions and answers. The third training session focused on preparation for the logistics of a Committee meeting. For this, Committee members participated in a mock committee meeting or observed an actual Community Review of MS Research Committee meeting.

## Execution

### Application review process

**Fig. 2:**
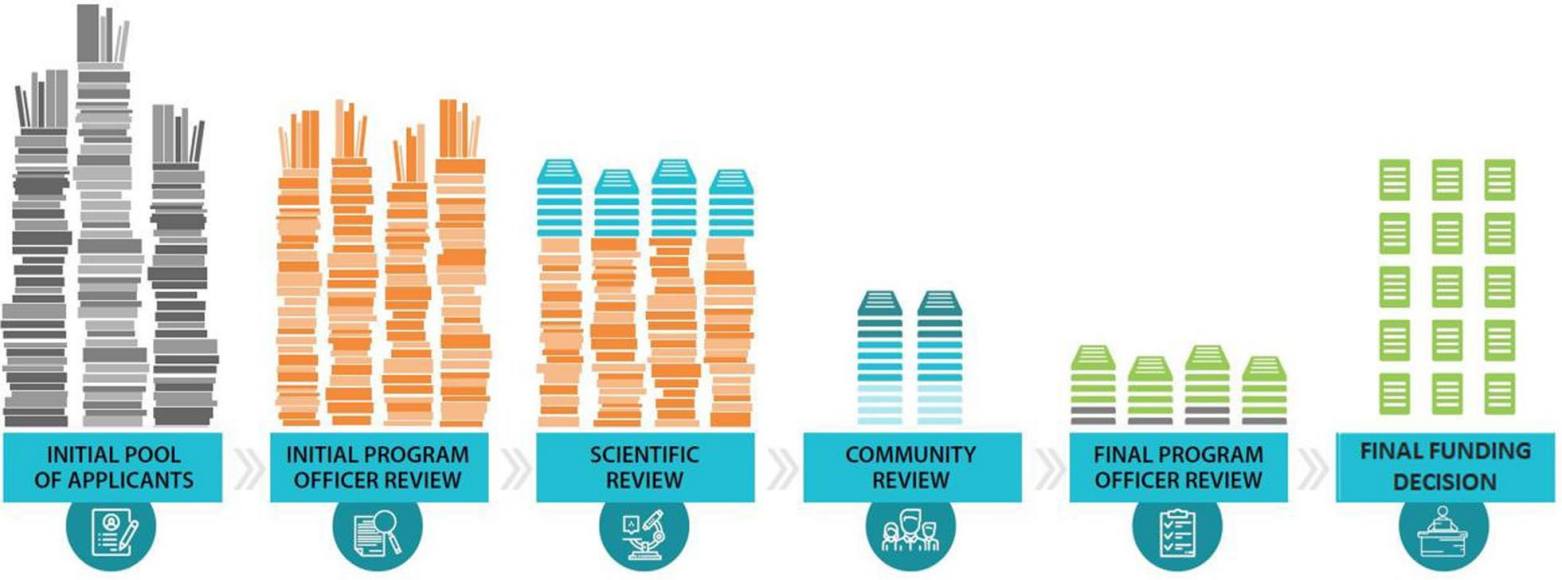
Overview of the multiple rounds of review applications submitted to the Society undergo before funding decision making

The creation of processes to review, score, and provide feedback on the applications was essential to the work of this new Committee and, per the goals initially set by the Community Engagement in Research Task Force, must align with the Society’s scientific review process. Committee members review all research grant applications submitted to the Society that are found to be scientifically meritorious; this includes two cycles per year. An optional cycle was added after the first year; in this third review cycle, research grant applications for commercial research funding through the Society’s Fast Forward program are reviewed. In the current review process used by the Society, applications submitted for funding consideration undergo multiple rounds of scientific review before being reviewed by the Committee (Fig. 2). Upon submission, applications are reviewed first by Society research staff, who are scientists with expertise in various fields related to MS, to ensure the application fits with the funding opportunity and has relevance to Society priorities. Applications are then reviewed by Society staff for content and assigned to the appropriate scientific experts for peer review. The applications undergo rigorous scientific review by outside experts who provide feedback to the Society on the scientific merit of each application. All research grant applications deemed to be scientifically meritorious remain eligible for funding and move on to the next round where they are reviewed by the Committee. The Committee provides feedback to the Society about patient-centered concerns and the relevance of the application’s proposed work to the MS community.

Although the purpose of the Committee is to provide the patient perspective and not to review the scientific merit of the applications’ proposed research projects, it was essential that the Committee members understand the basics of the project, including the goals, basic experimental design, and possible impact for the MS community to effectively provide feedback on patient-centered concerns and relevance to the MS Community. This presented a challenge that needed to be overcome before program implementation because members of the Committee intentionally do not have scientific backgrounds. This challenge was overcome using a multipronged approach. First, during the submission process applicants are required to provide a Plain Language Description of their application’s proposed research project. The Plain Language Description requires applicants to answer five questions (Table 4) that encourage applicants to provide a non-scientific summary of the application’s proposed project, including rationale, goals, aims, methods, timeline, and impact. Although the full application is available to Committee members, they are only required to read and review the Plain Language Description portion. Second, Society staff hold optional Office Hour sessions for Committee members to attend, in which application projects are summarized in plain language by Society staff, and any science-focused questions from the Committee members are answered. Additionally, Society staff are available via email or additional calls to answer any questions not addressed during the Office Hour sessions. This encourages Committee members to ask as many science-focused questions as possible before the formal review meeting when the discussion should be more focused on identifying patient-centered concerns and communicating the relevance of the applications’ proposed work to the MS community.

**Table 4:** Prompts that applicants answer to provide a non-scientific summary of the proposed project

Plain language description questions
What is the problem related to multiple sclerosis that you are addressing with this project? Please share your overall hypothesis(es) in plain language
How do you propose to address the problem? Please summarize your aims using plain language
Describe the steps that you are taking to achieve these aims. Please be specific about the experiments you are conducting, and whether studies involve humans (with cohort details such as age, race, gender, etc.), models of MS, or cell cultures (human or animal)
For studies that include people, please describe what is involved for participants in this study (number, frequency, and timing of visits; physical examinations; clinical measures; sample retrieval; imaging scans; symptom recording; etc.)
How might the results of this study potentially make life better for people affected by multiple sclerosis? Please include a time frame

Similar to the scientific review process, the Committee members independently review each application prior to the review meeting and submit preliminary scores (Table 5) and comments based on any identified patient-centered concerns and the relevance of the work to the MS community. Preliminary scores and comments are reviewed by Society staff to prepare for discussion during the Committee meeting. During the Committee meeting, all applications are discussed. Consistent with processes for scientific reviewers, if a Committee member has a real or perceived conflict of interest with a grant application such as a personal connection to or medical history with an applicant, that committee member recuses themselves from the discussion of that application. This same conflict of interest policy is used with the Society’s scientific peer review committees. The purpose of this policy is to avoid bias or the perception of bias in the Society’s funding decisions. Time is allowed for all Committee members to participate in the discussion and feel that they were heard, and their points considered, which is an important part of coming to a consensus. After discussion, all applications are binned into one of three groups (Table 6) based on consensus. Applications are not ranked, and there are no required quotas or percentages of the applications that need to be placed in any bin. The final binning and Committee comments are taken back to the Society’s Research team and are considered in combination with the scientific review committee feedback, Society budget, and funding priorities to make final funding recommendations to the CEO, who makes the final funding decisions. Importantly, all comments, both written and verbal, from the Committee members are summarized and provided back to the applicants (See examples Table 7). This allows applicants to better understand and consider the perspectives of the MS community in their future research projects.

**Table 5:** Description of the preliminary scoring scale used by Community Review of MS Research Committee members

Preliminary score definitions
1: Outstanding—Addresses a highly relevant question of priority interest to at least a segment of the MS community
2: Very Good—Addresses a very relevant question of interest to at least a segment of the MS community with only minor concerns
3: Good—Addresses a relevant question for the MS community but has at least one significant concern from a community perspective
4: Below Average—Addresses a question of limited relevance to the MS community and may have one or more concerns from a community perspective
5: Weak—Does not address a question of sufficient relevance to the MS community to warrant recommendation and/or has multiple significant concerns from a community perspective

**Table 6:** Description of the Bins that scientifically meritorious applications are placed in as the review outcome

Bin	Description
A	The top grant requests that have no patient-centered concerns and high relevance to the committee
B	The grant requests with a mixed response from the committee due to patient-centered concerns and/or moderate relevance
C	The grant requests that have significant patient-centered concerns and/or have limited relevance to the committee

**Table 7:** Examples of feedback provided by the Committee

General feedback category	Specific examples of feedback provided
Patient centered concerns regarding study burden to participants	Studies requiring multiple lumbar punctures of participants raised concern among Committee members as an excessive burden due to the potential side effects of the procedure
	Studies with a high number of or long required magnetic resonance imaging (MRI)sessions raised concerns of participant burden based on the discomfort MRIs can induce
	A requirement of frequent site visits by participants has raised concerns regarding the burden this would results in due to the amount and time required to arrange and undergo frequent travel and transportation to the site
Concerns regarding study participant compensation	Concerns are raised when it is not clear if or how participants will be compensated for their participation in studies, especially for studies that require frequent site visits or significant amounts of participant time
	A lack of information about reimbursement for travel and parking related expenses associated with study participation are common concerns raised by committee members
Question regarding diverse representation in the participant pools of proposed studies	Applications that lack information about the target or anticipated demographics of the study participation pool often raise concerns among the Committee because diverse representation in MS research is a priority of Committee members
	When applications share that their proposed research will have limited diversity in the sample pool due to an unavoidable limitation of the study, Committee members do express concern but are grateful that the applicant is aware of the limitations of their study and the current state of the field
Ensuring study participants are fully informed on the study, requirements, and risks	The Committee voices the important of informed consent and clear communication to participants of the risks when an application proposes a research project that is perceived as high risk by committee members
Concerns regarding the applicant’s ability to share the work proposed in their application in a lay friendly way	When applicants are unable to clearly explain their proposed work in the application plain language description, the Committee is likely to raise concerns based on the perceived ability of the applicant to explain their work clearly to those outside of the field, including study participants if applicable

The majority of Committee meetings have been held virtually with good success. Committee members were all able to resolve any technical issues and showed good engagement during the meetings. Meeting in person was intended to occur intermittently to encourage continued engagement and to mirror the scientific review meetings. An in-person meeting was held in 2023 when all Committee members were brought together for a day-and-a-half-long meeting to review applications for a single cycle. Holding the meeting in person brought both benefits and drawbacks in comparison to holding the meeting virtually. Planning and executing an in-person meeting required significant staff resources to coordinate the meeting venue, lodging, Committee member travel, catering services, as well as ensuring the travel, lodging, and meeting venue were accessible for Committee members. The in-person meeting also required more time commitment from Committee members as they had to travel to the meeting location and stay in the hotel where the meeting was held, with most Committee members staying at least two nights in the meeting location. This process also incurred more expenses for the Society, which covered all meeting associated costs, compared to meeting virtually. Feedback from survey data, following the in-person meeting revealed that 50% of respondents prefer in-person meetings, although there were comments about travel being difficult. One committee member wrote, “Traveling is tough, but I like in person meeting”. Approximately 75% of respondents report a preference for having an in-person meeting annually or once every other year, but several respondents said once was enough. Committee members shared an awareness of the cost of bringing everyone together. “I greatly appreciated that the Society paid for the travel for my care partner.” Overall, the in-person meeting was considered a success, and it is likely that additional in-person meetings will be held periodically, while still holding the majority of Committee meetings virtually.

### Committee member and staff workload

The number of applications reviewed and the bins they are assigned vary substantially cycle to cycle. Since its initiation, the Committee has reviewed a total of 116 applications and participated in an estimated 83 h of review meetings. Of the 116 applications that this Committee has reviewed, 76 have been recommended by the Committee for funding by the Society. After each cycle the Committee members are given a form to share the amount of time they spent preparing for the Committee meeting and in the Committee meeting. Review of the three cycles held in 2023 indicates that, on average, Committee members estimate the total amount of time they devote to this Committee, both individually and at review meetings, is about 1.6 h per application for a virtual meeting. However, the time required of Committee members for the single in-person meeting increased to 5.3 h per application, which is likely due to the required travel time. Time spent per application would likely decrease if the number of applications reviewed in person was larger.

For this Committee, the Society makes a substantial commitment which supports two research staff members and an administrator to oversee the program. The two staff are trained scientists on the research team and each dedicates an estimated 15% of their time to this committee to support an average of three review cycles a year allowing for an average of 35–40 applications to be reviewed each year. They co-lead the group, addressing scientific questions about the applications and working to build consensus, however, staff perspectives are not included in the binning process during the review meetings. The administrator also has other responsibilities in addition to their work with the Committee, which includes facilitating access to grant application via the Society’s secure grant portal and managing required committee paperwork. The Society has continued its relationship with the Kith Collective to provide feedback and guidance, help with quality improvements, assist with Committee training, support program management, and address changes in the Committee process as the Committee grows. The Committee members participate in the committee on a volunteer basis but are reimbursed for any expenses that occur due to participation, such as travel-related expenses. The same approach is used for the scientific and other volunteer committees at the Society. The Society has found this investment is sustainable due to the benefit it provides.

## Refinement

A key element in the evolution of the Committee has been refining the process over time to improve the transparency of the review process for the Committee and the clarity and accuracy of the Plain Language Descriptions from the applicants. To collect feedback from our Committee members, a survey was created in which Committee members are asked questions regarding how the cycle went for them and what improvements are needed (Table 8). Surveys provide feedback on the amount of time Committee members need to review the application, feedback on the office hours and review meetings, as well as a place for Committee members to provide other feedback to Society staff. Informal feedback is also solicited and received from Committee members during office hours and meetings as well as through email during and in between cycles.

**Table 8:** Sample survey questions answered by Committee members to identify areas of improvement

Question	Type of response
I felt that the training materials and sessions helped prepare me for the research review process	Six-point scale ranging from Strongly Disagree to Strongly Agree
Which training materials and exercises did you feel were helpful?	A check all that apply pick list containing examples of training materials and office hour sessions
Do you use a dictionary or glossary that might be a helpful resource for others on the committee? If so, please specify what you use	Yes/No selection with open ended option if yes
I felt that the plain language descriptions written by applicants were useful for scoring	Six-point scale ranging from Strongly Disagree to Strongly Agree
The time required to read through and rate the applications prior to the meeting was manageable	Six-point scale ranging from Strongly Disagree to Strongly Agree
Was the window of time given to review the grant applications appropriate?	A select one pick list of Yes, No (a longer review period would be preferable), or No (a shorter review period would be preferable)
I had adequate opportunity to ask questions prior to the committee meeting	Six-point scale ranging from Strongly Disagree to Strongly Agree
The time required to reach consensus and bin the applications at the committee meeting was manageable	Six-point scale ranging from Strongly Disagree to Strongly Agree
I had adequate opportunity to express my thoughts at the committee meeting	Six-point scale ranging from Strongly Disagree to Strongly Agree
I feel that my opinions were heard and that my perspectives were respected	Six-point scale ranging from Strongly Disagree to Strongly Agree
My interactions with Society staff were positive	Six-point scale ranging from Strongly Disagree to Strongly Agree

Feedback from committee members about their experience has been very positive: “I like being able to share my voice and lived experience to impact how the society allocates research funding, continued learning about the future of MS treatment/symptom management efforts, and of course the amazing cohort of people on the committee,” “I like “Having an opportunity to share my perspective and hear the perspectives of others affected by MS on potential MS research.” In addition, based on the feedback received over time, refinements to the Committee’s review processes continue to be made. For example, the prompts in the application for the Plain Language Description have been updated over time to elicit more lay friendly responses while maintaining enough detail for the Committee members to fully review the application. There were only positive responses to this change from committee members, for example, “It is wonderful to see the applicants not only taking the plain language description request seriously but overall, making a definitive and successful effort to write them.” Additionally, based on the Committee members’ feedback, the format of the Office Hours has been updated. The Office Hours originally had a question-and-answer format where Committee members were expected to bring their questions to the call to be answered by Society Research staff. However, after receiving positive feedback that including a short summary of each application would help spur conversation, the process has been updated to have the first Office Hour focus on the delivery of short summaries of each research application by Society Research Staff. Later Office Hour sessions are then formatted as question-and-answer sessions. Both refinements were made to enhance the understanding of scientific concepts by the Committee members, so they are comfortable providing insights about these applications from their lived expertise in MS. Comments from the committee in response to these changes were very positive: “I really appreciate the first session summarizing the applications,” “I like and appreciate the first office hours going through each proposal with the other hours being a Q/A.”

Additional process improvements have been made to improve Committee engagement and optimize the Committee outcomes. For example, after the first year of virtual Committee meetings, meeting durations were shortened from five to four hours on each day to allow the Committee to stay engaged throughout the meeting. Initial virtual meetings were observed to be longer than necessary, with long meetings resulting in decreased engagement toward the end of the meeting. The shorter meeting duration and improved Committee member engagement encourages more productive conversations and more effective application binning. The language defining the final bins (Table 6) that the applications are placed into by the Committee has also been updated over time based on Committee member feedback. By updating and clarifying the language used to describe the criteria for each bin, the Committee is able to come to consensus and bin each application more efficiently. Finally, the feedback provided to the applicants has changed over time to be more actionable for the applicants. Currently, feedback provided to the applicants focuses on key themes from the Committee member conversations, relaying Committee member rationale for their comments and including actionable suggestions the applicant could incorporate in future submissions.

Committee turnover is an important component of the Committee and similar to the Society’s Scientific Review Committees, the turnover process ensures that there are diverse community perspectives and viewpoints included. After a full year of review cycles with the inaugural Committee, a turnover process was developed where approximately one-third of the Committee rolls off and is replaced with members who are new to the Committee. To accomplish this turnover, engagement conversations are held with Committee members to help gauge the fit of the Committee for each volunteer. During engagement conversations we address the members’ overall experience so far, we explain the roll-off process, we ask the committee member if they would like to roll off or stay for another year, and we invite them to think about friends who might be interested in having a similar experience. We explain the nomination process and encourage the committee members to reach out to friends and Society staff if they have suggestions for new member nominations. Roll-off decisions are made using information from those conversations as well as reflections on the need to represent the diverse aspects of the MS population. New members joining the Committee are selected using the same process developed for initial implementation, with Society staff members submitting nominations for possible members who have experience volunteering with the Society. The nominated individuals are interviewed and selected based on their interest in the work of the Committee and Committee needs. It is anticipated that as this yearly roll off/on process continues, the Committee will establish cohorts of Committee members who roll on the Committee and serve for a three-year term before rolling off, with continued yearly engagement conversations to ensure that the Committee continues to be a good fit for the volunteer. This will establish a regular and predictable term for Committee members and maintain a steady influx of new ideas and MS community perspectives within the Committee.

## Discussion

Engaging community reviewers in research funding decision-making is not new, however, the Society has taken steps to integrate the MS community more fully into the process in a way that has not been shared publicly until now. The new Community Review of MS Research Committee features several unique and important differences from the process used previously and used by many other funders.

1. The Committee meeting does not include external scientists or technical experts. The Committee meetings are designed for open discussion among Committee members with no scientists to which to defer. This allows for a focus on patient-centered concerns and relevance of the application’s proposed work to the MS community. Questions related to the scientific aspects of the applications are encouraged at the Office Hour sessions in the weeks before the Committee meeting. In these Office Hour sessions, Society staff guide the discussion of scientific concepts that are not well described in the plain language summary and necessary to assess patient-centered concerns and relevance to the MS community. By addressing these questions prior to the review meeting, the discussion during the review meeting stays focused on identifying patient-centered concerns and relevance of the applications’ proposed work to the MS community.
2. To streamline the work and time required, the Committee only reviews the grant applications that have been deemed scientifically meritorious by the scientific review committee. This limits the number of applications the Committee is asked to review and ensures that they are reviewing applications with strong scientific merit and rigor. The scientific review committee first filters out the applications that are not scientifically ready to be executed, regardless of funding availability. The order of these two reviews is intended to be as inclusive as possible of the Committee while respecting their time, effort, and expertise.
3. The consensus process used to bin the applications during the Committee meeting is also unique to this committee. This process is separate from the preliminary score that is submitted online by each Committee member before the Committee meeting, from their independent work. The consensus process used for binning is intended to allow the Committee the opportunity to consider all Committee member’s feedback and concerns about the application, ensuring that all feedback is heard before coming to a group consensus. The applications are binned, not ranked against each other, and there is no quota for the maximum or minimum number of applications placed in each bin. The bin in which each application is placed is used by Society staff during final funding decisions.

The creation of this new process and formation of this Committee has the potential to provide multiple benefits to the Society, the MS community, and the members of the Committee. A future step in this work is to track the impact of this committee’s work. As a whole, the Society receives systematic feedback from members of the MS community that affects funding decision-making. This Committee also creates a space in which Committee members can inform the Society about what is important to the MS community. Already the society staff who worked to create and run this new Committee have learned some of the issues that are important to the MS community. Bringing these learnings to the broader Society team has allowed for the work done by the Society to be better informed about topics that are relevant to the MS community. For example, feedback from this Committee is now requested as the Society develops calls for applications and their feedback contributed to the decision to focus a call for applications on generating knowledge and tools to promote remyelination and neuroprotection in MS in the fall of 2023. Additionally, this Committee has increased Society Staff awareness of gaps in how scientists explain their work and has improved the staff’s abilities to translate complex scientific concepts to a non-science-trained layperson. More benefits to the Society are likely to continue to emerge as this Committee conducts future review cycles.

In addition to benefits to the Society, the MS community and broader research community may also benefit from this newly formed Committee. The Committee provides feedback on the research being conducted and funded. This feedback not only impacts the work that is funded by the Society but also provides feedback to the scientists conducting the research. By receiving and responding to feedback from the MS Community, the research environment may be more focused on addressing high priority issues for the MS community in ways that are not overly burdensome to those living with MS. It is anticipated that this focused approach will more efficiently address the needs of those affected by MS, increase the quality of the work conducted, increase community trust, and promote ethical and thoughtful research practices.

Finally, there is also a benefit to the members of the Committee, which is noted from comments to Society staff receive and those written into the end of cycle surveys. A few example comments include, “The combination of both learning about and discussing the research in the company of the MS community represented on the committee is an incredible experience,” and “I like reading the grant applications and seeing the progress being made towards a cure for MS. I enjoy participating in the office hours and the consensus meetings hearing other committee members thoughts and opinions”. The Committee members themselves report that they have gained immensely from their experience on the Committee. The benefits reported include learning how the research process works and how Society funding fits into the greater research landscape. Committee members have also reported that their experience makes them ambassadors for the Society in teaching others in their community about how the Society funds research and what the Society focuses on. Committee members overall report enjoying the process of being on the Committee and express enthusiasm to stay on the Committee.

Despite the many benefits of the new process, as with all new endeavors, several challenges were faced in the formation of this Committee and the solutions used to overcome these challenges deserve description here. Some challenges of note include achieving a diverse Committee that represents the MS community as much as possible with a Committee of 14 people, ensuring that Committee members are aware of all types and stages of research and can appreciate the importance of all types of research paradigms, and encouraging high quality, readable Plain Language Description submissions from applicants. These challenges were identified early in the development of this Committee and strategies were developed to overcome the challenges and create a successful process. Although strategies are being employed to address these challenges, the Committee and its process continue to evolve to better address these and new challenges that arise.

The importance of diversity in this Committee is clear because the Committee is intended to represent the MS community, thus a top priority was for the Committee to be as diverse as the MS community itself. The challenge was to create a committee that is diverse in not only gender, race, and ethnicity, but also diverse in terms of a person's journey with MS, age, geographic location, past experience with research, and level of physical ability among others. These types of diversity are critical on the Committee because they shape a person’s perspective and therefore impact the MS community, but it is challenging to gather this information comprehensively. To address this desire to recruit a diverse Committee, the nomination process includes a teleconference conversation with two Society research staff allowing the time to hear details about each nominee’s journey with MS and their unique perspectives from past volunteer experiences. This approach is used to assure that each nominee feels heard, valued, and respected for their lived expertise in MS. After holding conversations with all nominees, a discussion of the unique perspectives each nominee brings is held between multiple Society staff and consultants from the Kith Collective before Committee members are selected to ensure that the Committee is as inclusive and diverse as possible. The committee was also designed with accessibility in mind and intentionally is composed of those with a range of physical abilities and types of MS symptoms. However, it is recognized that participation on the committee may present a burden to some members of the MS community. Attempts have been made, when possible, to overcome those burdens, including relying primarily on virtual meetings that are structured to keep committee members as comfortable as possible and providing printed, in addition to electronic, materials for participants who find that more accessible. Working with individual committee members to find accommodations that work for them has allowed those with more significant physical challenges to participate in the committee.

Thus far the committee has been composed of approximately 42–43% men with 14% of the committee members representing MS care partners. While full committee member demographics are not currently shared to preserve committee members’ confidentiality, there is a goal to track demographics over time to ensure diverse representation on the committee and work to continue to make the committee as accessible as possible. Despite the efforts to create a diverse committee, limitations do remain and continue to be addressed. As the committee matures there are goals to include additional dimensions of diversity that have thus far been difficult to include such as committee member level of education, socioeconomic status, those recently diagnosed with MS, young adults living with MS, those with different approaches to risk or familial aspirations, those with the highest levels of symptoms, and other groups of people affected by MS who may find participation on the committee less accessible. There is also a risk that the nomination process introduces biases to the committee, as committee members must have past experience engaging with the Society. The nomination process helps Committee staff ensure that committee members will reliably and responsibly engage with the Society and, thus, is critical to Committee recruitment. However, a wide range in the type of past engagement with the Society is sought when recruiting nominations in an effort to increase the diverse experiences brought to the committee by its members.

A second challenge was to ensure that Committee members were aware of and could appreciate all types of research paradigms. The Committee would be reviewing applications that cover all aspects of the research landscape, from foundational science to clinical trials, rehabilitation and science policy. There were concerns raised that the Committee may consciously or unconsciously be biased toward supporting specific types of research over others, for example favoring clinical trials over basic research or vice versa. The Society believes that funding research at all stages is critical to achieving cures for MS, therefore it was important that the Committee also has this appreciation for the importance of all stages of research. This was addressed during Committee member recruitment, where potential Committee members were made aware of the breadth of the research landscape that the applications could cover and that the Committee members would be expected to review. This was discussed with the nominees during the interview process where any questions, concerns or points of view from the nominee were answered by Society staff. The inclusion of this information during the recruitment stage ensured that new Committee members came in with an awareness of the breadth of research the Society reviews. Further, regular additional training is provided to the Committee to continuously reinforce this idea and the importance of consensus with established Committee members. Through these strategies, the Committee has thus far not exhibited an observable bias for or against any type or stage of research and staff remain vigilant about the need for additional contextualization and training to support committee members' understanding of the importance of various types and stages of medical research.

A third challenge was how to encourage research grant applicants to write an accurate and readable Plain Language Description of their application’s proposed project. High quality and lay-friendly Plain Language Descriptions submitted by applicants are critical to the success of this Committee because the Committee relies heavily on the Plain Language Description for the review of each application. As was described previously, Society staff composed five questions designed to lead the applicant to share the important elements of their application’s proposed work in plain language terms. Results from initial cycles were mixed, with some applicants being more effective at writing Plain Language Descriptions than others. To overcome this challenge, Society staff continue to modify the five questions asked of applicants in the Plain Language Description to be as clear as possible and Staff provides written feedback from the Community Review of MS Research Committee to the applicant after each cycle. Over the last 2.5 years, improvement has been seen in the Plain Language Descriptions overall, which has been commented on by the Committee members. Despite this overall improvement, there are exceptions with new and returning applicants. The Society acknowledges that in general, researchers are not trained to write in a lay-friendly way, and therefore the applicants have differing approaches, styles, and skills when drafting their Plain Language Description. There is growing recognition of the essential need to articulate science in clear terms that can be understood by a wide range of stakeholders. To address this issue at its root the Society has implemented new steps in the process: (1) When applicants request to discuss review comments with Society staff, the staff discuss the Committee comments as well as the scientific review comments over individual teleconference meetings; (2) The Society has started providing sessions on scientific communication at its biennial Tykeson Fellows Conference, a conference that all Society funded fellows, trainees, and junior faculty awardee attend. It is anticipated that this will continue to be a challenge and will require time and repetition for the applicants to become accustomed to the writing requirement of the Plain Language Description.

The creation and maintenance of this Committee has resulted in several learnings that have been critical to the success of this work.

1. The Committee started with the focus of integrating the MS community more completely into the Society’s research decision-making process. Substantial progress has been made in doing this, and that has required a significant investment of staff time and resources. The investment of two staff members leading the Committee has proven to be important for the discussion of ideas and issues in real-time as they arise. Having two staff members equally informed and invested brings different perspectives to the planning process, the Committee itself, and the response to questions/concerns Committee members share throughout the year. The intentional recruitment process also takes time to gather information from Committee members and Society staff but assures that both groups are well informed.
2. Another learning has been the value of training Committee members in how the Society assesses research grant applications, Committee members have shared that this information is of interest to the MS community. It has been observed that Committee members serve as informal “ambassador volunteers” by sharing the Society’s grant review process.
3. The importance of consensus building and engaging experts in patient-centered practices has been critical to the success of the new Committee. By engaging the Kith Collective, a consulting practice dedicated to speeding adoption of patient-centered practices that return lasting social value, the Society was able to intentionally create a patient-focused committee that uses consensus building to reach final recommendations that guide funding decisions. Novel insights from the Kith Collective during Committee planning, communication, recruitment, training, and maintenance have proven to be vital to the success of this Committee.
4. Establishing and institutionalizing process change requires dedicated time and attention. The Committee was a new undertaking for the Society and the need for external support was recognized early on in the development of the Committee. At the beginning of this process, the specialized experience provided by the Kith Collective helped the Society understand and assess the strengths and drawbacks of various approaches to incorporating community perspectives in research funding decisions. They helped articulate the key objectives in undertaking this process change and designing an approach to meet the Society’s specific objectives. In the early stages of implementation, the Kith Collective helped with continuous process improvement and refinement of each step toward convening the Committee, preparing members for the research review task, and assimilating members' individual and collective input. As the Committee has matured, the Kith Collective provides ongoing program management support to supplement time dedicated by internal staff.
5. Finally, the importance of consistent process reevaluation and evolution was observed during the maintenance of the Committee. Twice a year, Committee members share feedback with the Society through a survey. This allows Society staff to hear thoughts and concerns from the Committee members and make process improvements as a result. Multiple improvements have been made to the Committee processes based on feedback from these surveys including meeting length, format of the Office Hour sessions, and Committee training. Society staff also directly address concerns of individual Committee members in a 1:1 setting through annual engagement conversations and on an ad hoc basis.

In conclusion, the Committee was formed in 2021 and has thus far completed more than 8 cycles of review of research grants. The development of this Committee has allowed for learnings and key takeaways for the Society (Table 9). Through this Committee, as anticipated, it was clearly demonstrated that people affected by MS have important perspectives and insights into research projects focused on MS that are distinct from scientific peer reviewers. The formation of this Committee has allowed the Society to comprehensively and systematically incorporate these perspectives in research grant funding decisions. This article outlines the steps and strategies that have been employed in the planning, implementation, execution, and refinement of this Committee, which have been essential to its success. This Committee has provided benefits to the Society, the MS community, and the Committee members. Challenges were encountered during the implementation and maintenance of this Committee, and these challenges have been overcome with careful planning and refinement of processes over time. We highlight an important future goal of tracking the impact of this committee on the Society and its decision-making process. The Committee will continue to evolve as new challenges and opportunities arise and amid the evolving needs of people living with MS. The Committee is one important element aiding the Society to achieve its mission of curing MS while empowering people affected by MS to live their best lives. The information shared here is intended to be useful for other pathology communities to use and apply so that community stakeholders have an opportunity to contribute to scientific research solutions.

**Table 9:** Key takeaways after from the National MS Society’s Community Review of MS Research Committee

Key takeaways
The Committee allows the Society to comprehensively and systematically consider the perspectives of people affected by MS when making research grant funding decisions
People affected by MS have important perspectives and insights into research projects focused on MS that are distinct from scientific peer reviewers
Creation of an environment that is centered on the MS community, without scientific peer reviewers, encourages Committee members to speak freely and share their point of view without deferring their valuable lived expertise to a trained scientist and researcher
The MS community is eager and willing to be integrated into the research grant review process
The consensus-building approach is an effective way to ensure all committee member perspectives are valued and honored and to determine which grants are of greatest interest to the MS Community
Applicants have differing abilities to clearly explain their proposed research in general language and continued education is needed for researchers to improve these skills

## Data Availability

No datasets were generated or analysed during the current study.

## Abbreviations

MS: Multiple Sclerosis
The Society: The National Multiple Sclerosis Society
The MS Community: People living with MS, care partners, family members, and others who are impacted by MS
The Committee: The Community Review of MS Research Committee
Scientifically meritorious: Description of applications that have undergone scientific review and were deemed ready for funding if sufficient funds were available
Office hours: Question and answer sessions held for Committee members by Society staff where summaries of application proposed research is shared and science-related questions from Committee members are answered by staff.
Consensus: A state of mutual agreement among members of a group where all legitimate concerns of individuals have been addressed to the satisfaction of the group (Saint & Lawson, 1994). Consensus does not require unanimity. It does require that each person has the opportunity to express concerns and that those concerns heard, understood and considered by group members.
Bins or Binning: Categories (A–C) in which applications are placed based on the Committee’s enthusiasm about the relevance of the application to the MS community and any identified patient-centered concerns. The applications are not ranked against each other and there is no requirement for a minimum or maximum number of applications placed in each bin. Descriptions of the bins can be found in Table 4.
Application: An application submitted to the National MS Society to request funding of a research project, especially through a research grant funding opportunity (investigator initiated or request for applications). The application includes a plain language description that is submitted by the applicant and explains the proposed research project in lay terms.
Grant: A grant or research grant is a funding mechanism provided by the National MS Society to successful applications. In this publication, grant refers to a research grant (not including fellowships and training awards), including investigator initiated or request for applications mechanisms.
